# Purine Metabolites in Tumor-Derived Exosomes May Facilitate Immune Escape of Head and Neck Squamous Cell Carcinoma

**DOI:** 10.3390/cancers12061602

**Published:** 2020-06-17

**Authors:** Nils Ludwig, Delbert G. Gillespie, Torsten E. Reichert, Edwin K. Jackson, Theresa L. Whiteside

**Affiliations:** 1 Department of Pathology, University of Pittsburgh School of Medicine, Pittsburgh, PA 15213, USA; nils.ludwig@ukr.de; 2 UPMC Hillman Cancer Center, Pittsburgh, PA 15213, USA; 3 Department of Oral and Maxillofacial Surgery, University Hospital Regensburg, 93053 Regensburg, Germany; torsten.reichert@klinik.uni-regensburg.de; 4 Department of Pharmacology and Chemical Biology, University of Pittsburgh School of Medicine, Pittsburgh, PA 15261, USA; dgg3@pitt.edu (D.G.G.); edj@pitt.edu (E.K.J.); 5 Departments of Immunology and Otolaryngology, Pittsburgh, PA 15213, USA

**Keywords:** exosomes, extracellular vesicles, TEX, HNSCC, head and neck cancer, purine metabolites, adenosine, purinergic signaling

## Abstract

Body fluids of patients with head and neck squamous cell carcinoma (HNSCC) are enriched in exosomes that reflect properties of the tumor. The aim of this study was to determine whether purine metabolites are carried by exosomes and evaluate their role as potential contributors to tumor immune escape. The gene expression levels of the purine synthesis pathway were studied using the Cancer Genome Atlas (TCGA) Head and Neck Cancer database. Exosomes were isolated from supernatants of UMSCC47 cells and from the plasma of HNSCC patients (*n* = 26) or normal donors (NDs; *n* = 5) using size exclusion chromatography. Ultraperformance liquid chromatography–tandem mass spectrometry (UPLC-MS/MS) was used to assess levels of 19 purine metabolites carried by exosomes. In HNSCC tissues, expression levels of genes involved in the purinergic pathway were upregulated indicating an accelerated purine metabolism compared to normal tissues. Exosomes from supernatants of UMSCC47 cells contained several purine metabolites, predominantly adenosine and inosine. Purine metabolite levels were enriched in exosomes isolated from the plasma of HNSCC patients compared to those isolated from NDs and carried elevated levels of adenosine (*p* = 0.0223). Exosomes of patients with early-stage disease and no lymph node metastasis contained significantly elevated levels of adenosine and 5′-GMP (*p* = 0.0247 and *p* = 0.0229, respectively). The purine metabolite levels in exosomes decreased in patients with advanced cancer and nodal involvement. This report provides the first evidence that HNSCC cells shuttle purine metabolites in exosomes, with immunosuppressive adenosine being the most prominent purine. Changes in the content and levels of purine metabolites in circulating exosomes reflect disease progression in HNSCC patients.

## 1. Introduction

Head and neck squamous cell carcinomas (HNSCC) account for about 40,000 new cases per year in the United States and more than 600,000 cases annually worldwide [1]. Only 40–50% of patients with HNSCC will survive for five years and this is in part a result of the different etiologies and the heterogeneity of molecular changes that drive carcinogenesis of HNSCC [1,2]. Most epithelial malignancies, including HNSCC, are characterized by overexpression of oncogenes, growth factor receptors, enzymes and various immunosuppressive factors inhibiting functions of immune cells [2,3]. One important mechanism facilitating tumor escape from immune surveillance is the signaling via purine nucleotides and nucleosides, such as adenosine and adenosine 5′-triphosphate (ATP) [4]. Purinergic signaling plays a key role in modulating immune responses in physiological and pathological conditions. Physiologically, anti-inflammatory effects of adenosine balance proinflammatory ATP, protecting tissues from damage caused by activated immune cells. Pathologically, increased adenosine monophosphatase (AMPase) activity in tumors leads to increased adenosine production, generating a deeply immunosuppressed tumor microenvironment (TME) and promoting cancer progression [5].

Most recently, tumor-derived exosomes (TEX) have been recognized as one of the key immunosuppressive mechanisms operating in the TME [6]. These virus-size (30–150 nm) extracellular vesicles (EVs) are mediators of intercellular communication and serve as the transport system for a variety of molecules and factors shuttled between the tumor and immune cells locally in the TME and at distant sites [7]. The composition of TEX cargos is complex and includes the cell membrane-derived as well as cytosolic components of the producer tumor cell, including a broad variety of proteins, lipids and nucleic acids [8]. Surface-bound ligands on TEX are competent in inducing signaling-cascades in recipient cells, which leads to functional reprogramming of cells in the TME and promotes the formation of the immunosuppressive tumor-associated immune system [9]. Besides surface interactions, TEX are internalized by recipient cells, delivering nucleic acids and initiating the generation of new functional proteins [10]. Recent evidence suggests that TEX may interact with recipient cells via purinergic signaling pathways [11]. Specifically, our group has previously shown that HNSCC-derived exosomes carry components of the adenosine pathway, including the ectonucleotidases CD39 and CD73 [11,12]. These enzymes were functionally-active, as measured by exosome-mediated hydrolysis of exogenous (e)ATP to adenosine, and were present in exosomes isolated from the plasma of normal donors (ND) or HNSCC patients [12]. Additional analysis indicated that exosomes obtained from the plasma of HNSCC patients had higher levels of ectonucleotidases than exosomes isolated from the plasma of NDs [12]. Additionally, Clayton et al. [13] reported that 5′-AMP converted to adenosine by CD39^+^/CD73^+^ TEX inhibited T cell activation via the adenosine A_2A_R independently of CD73 expression on T cells.

While the presence of ectonucleotidases on the exosome surface has been well documented, little is known about the presence of other components of the purinergic signaling pathway in the exosome cargo. Sayner et al. [14] reported that exosomes derived from pulmonary microvascular endothelial cells as well as exosomes collected from the perfusate of isolated rat lungs contained cAMP that mediated cellular signaling. Our recently published data indicated that TEX produced by an HNSCC cell line contained adenosine and inosine and induced adenosine A_2B_R-mediated responses in recipient endothelial cells [11]. These data suggested that exosomes released by tumor cells might encapsulate various components of the purinergic pathway. To further investigate the presence and role of purine metabolites in HNSCC-derived exosomes, we screened exosomes isolated from supernatants of HNSCC cell lines and from the plasma of HNSCC patients for a range of purine metabolites by mass spectrometry. We found that TEX were enriched in purine metabolites and that a correlation between levels of these metabolites in TEX and clinicopathological endpoints in HNSCC patients could have clinical significance.

## 2. Materials and Methods

### 2.1. The Cancer Genome Atlas (TCGA) Analysis

The TCGA Head and Neck Cancer database was analyzed using the University of California, Santa Cruz (UCSC) Xena Browser [15]. In total, 520 cases of primary HNSCC were included in this study. Tumors were resected from different locations across the patients’ heads and necks and represented stage I to IV tumors. Data was compared to 44 “solid tissue normals” which are taken from normal tissues near the tumor. Gene expression data of defined gene sets were generated and clustered online, and the data were downloaded for subsequent statistical analysis.

### 2.2. Cell Lines

The HPV(−) cell lines PCI-13 and PCI-30 derived from human HNSCC were established and maintained in our laboratory [16] and the HPV(+) cell lines UMSCC2, UMSCC47 and UMSCC90 were established by Dr. Thomas Carey (University of Michigan, Ann Arbor, MI, USA) and obtained from Robert L. Ferris (UPMC Hillman Cancer Center, Pittsburgh, PA, USA). Given the equivalent exosome sample purity and comparable results within the cell lines, UMSCC47 cells are presented as a representative line [17]. The detailed characteristics of TEX deriving from HPV(+) or HPV(−) HNSCC cell lines were previously described by us in detail [18,19]. Cells were authenticated prior to their use and were grown in DMEM (Lonza Inc., Basel, Switzerland) supplemented with 1% (*v*/*v*) penicillin/streptomycin and 10% (*v*/*v*) FBS (Gibco, Thermo Fisher Scientific) at 37 °C and in the atmosphere of 5% CO_2_ in air. FBS was depleted of exosomes by ultracentrifugation at 100,000× *g* for 3 h. For exosome isolation, 2.5 × 10^6^ UMSCC47 cells were seeded with 25 mL media in 150 cm^2^ cell culture flasks as previously described [17]. Supernatants were collected after 72 h.

### 2.3. Patients

Peripheral venous blood specimens were collected from 26 patients with HNSCC with active disease (AD) seen at the UPMC Otolaryngology Clinic in 2016–2019. Blood specimens from 5 normal donors (NDs) served as controls. Informed consent from all individuals was obtained, and the study was approved by the institutional review board of the University of Pittsburgh (UPCI 09-069/IRB991206, annually approved, last date of approval: 7 April 2020). The blood samples were delivered to the laboratory and were centrifuged at 1000× *g* for 10 min to separate the plasma from blood components. Plasma was stored in 2 mL aliquots at −80 °C and thawed just prior exosome isolation.

### 2.4. Exosome Isolation by mini-SEC

The processing of supernatants and exosome isolation by mini-SEC was performed as previously described in detail [20]. Briefly, cell culture supernatants were centrifuged at room temperature (RT) for 10 min at 2000× *g*, transferred to new tubes for centrifugation at 10,000× *g* at 4 °C for 30 min and filtrated using a 0.22 µm bacterial filter. Afterwards, aliquots of supernatants were concentrated by using Vivacell 100 concentrators (Sartorius, Göttingen, Germany) at 2000× *g*. 1 mL of concentrated supernatant was loaded on a 10 cm-long Sepharose 2-B column (Bio-Rad, Hercules, CA, USA) and individual 1 mL fractions were collected. Fraction #4 containing nonaggregated exosomes was used in subsequent assays.

For isolation of exosomes from patients’ or NDs’ plasma, cryopreserved samples were thawed, centrifuged at RT for 10 min at 2000× *g*, transferred to new tubes for centrifugation at 10,000× *g* at 4 °C for 30 min and filtrated using a 0.22 µm bacterial filter. Afterwards, 1 mL of precleared plasma samples were loaded on individual Sepharose 2-B columns as previously described [20].

### 2.5. Exosome Characterization

Determination of protein concentrations by BCA, transmission electron microscopy (TEM) and Western blotting of isolated exosomes was performed as previously described [20]. Size distribution and concentrations of the particles were analyzed using tunable-resistive pulse sensing (TRPS) by qNano (Izon, Medford, MA, USA) as described previously [21]. The methodology was optimized according to current MISEV2018 guidelines from the International Society for Extracellular Vesicles [22].

### 2.6. UPLC-MS/MS for Purine Metabolites

Hundred µg of exosomes in PBS were mixed with internal standards and injected onto a Waters (Milford, MA, USA) Acquity ultraperformance liquid chromatograph (UPLC) connected to a Waters UPLC BEH C18 column (1.7 µm beads; 2.1 × 150 mm). Purines were quantified by selected reaction monitoring using a triple quadrupole mass spectrometer (TSQ Quantum-Ultra; Thermo Fisher Scientific, San Jose, CA, USA) with a heated electrospray ionization source. The mobile phase was a linear gradient flow rate (300 μL/min) of 1% acetic acid in water (pH, 3; mobile phase A) and 100% methanol (mobile phase B). The gradient (A/B) settings were: from 0 to 2 min, 99.6%/0.4%; from 2 to 3 min, to 98.0%/2.0%; from 3 to 4 min, to 85.0%/15.0%; from 4 to 6.5 min, to 99.6%/0.4%. The instrument parameters were: sample tray temperature, 10 °C; column temperature, 50 °C; ion spray voltage, 4.0 kilovolts; ion transfer tube temperature, 350 °C; source vaporization temperature, 320 °C; Q2 CID gas, argon at 1.5 mTorr; sheath gas, nitrogen at 60 psi; auxiliary gas, nitrogen at 35 psi; Q1/Q3 width, 0.7/0.7 units full-width half-maximum; scan width, 0.6 units; scan time, 0.01 s. The mass transitions, collision energies and retention times for the measured purines and their corresponding internal standards are provided in Table 1 and Table 2.

### 2.7. Statistical Analysis

All data were analyzed using the GraphPad Prism software (v7.0). Values are expressed as mean ± SEM. Differences between groups were assessed by Student *t* test and differences were considered significant at *p* < 0.05.

## 3. Results

### 3.1. mRNA Transcripts for Purine Metabolites Are Upregulated in HNSCC

**Figure 1:**
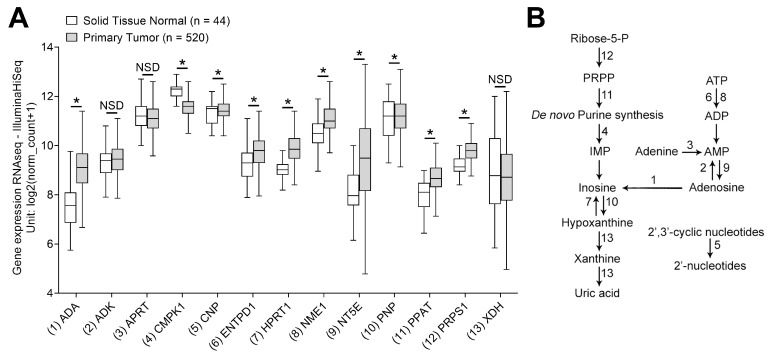
Analysis of gene expression levels of individual purine synthesis pathway mRNAs in the Cancer Genome Atlas (TCGA) database for head and neck squamous cell carcinoma (HNSCC) using the University of California, Santa Cruz Xena Browser. (**A**) Gene expression levels in 520 cases of primary HNSCC were compared to 44 solid normal tissues. Box and whiskers plot showing median, range of 25–75% and the minimum–maximum values. * *p* < 0.05. (**B**) A schema showing associations between purine metabolites. Numbers correspond to *x*-axis labels in (**A**) and indicate functions of enzymes that were analyzed using the TCGA database.

To characterize the impact of purine metabolism in HNSCC, the gene expression levels of a selected panel of mRNAs involved in purine synthesis were analyzed using the TCGA database. The associations between purine metabolites are presented in Figure 1B. Gene expression levels in 520 cases of HNSCC were compared to the expression levels in 44 samples of nonmalignant solid tissue. The analysis revealed that most genes involved in the metabolism of purines were elevated in expression in HNSCC compared to normal tissues. In particular, levels for *ADA*, *ENTPD1*, *HPRT1*, *NME1*, *NT5E*, *PNP*, *PPAT* and *PRPS1* were all significantly elevated in HNSCC tissues (*p* < 0.0001; Figure 1). In contrast, there was a significant downregulation of *CMPK1* and *CNP* in HNSCC tissue compared to normal controls (*p* < 0.05; Figure 1A). No statistical differences were observed for *ADK*, *APRT* and *XDH*.

These data indicate that HNSCC tissues require and utilize higher purine levels, and that this demand for purines is met by the upregulation of the purine metabolism in cancer cells. We hypothesized that as a consequence of the higher production of purine metabolites in tumor cells, exosomes produced by these cells are also likely to be enriched in various purines.

### 3.2. TEX Produced by HNSCC Cells Encapsulate Purine Metabolites

**Figure 2:**
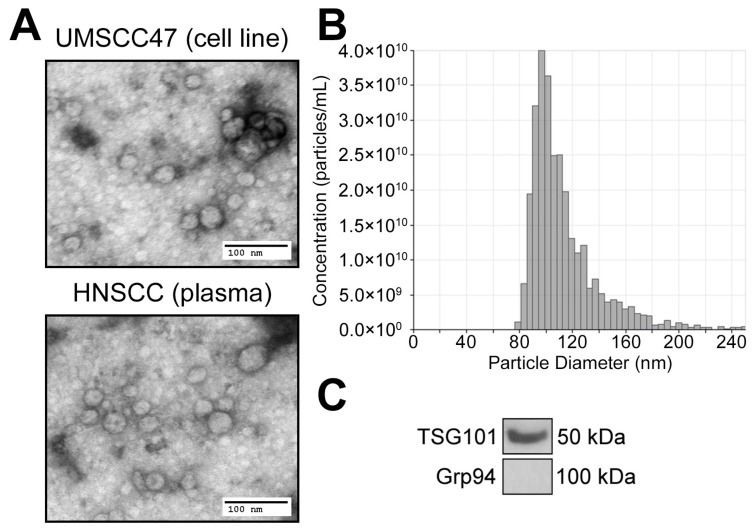
Characterization of exosomes from the supernatant of HNSCC cells or patients’ plasma. (**A**) Representative TEM images of UMSCC47-derived exosomes (upper panel) and exosomes isolated from the plasma of an HNSCC patient (lower panel). (**B**) Representative TRPS (qNano) size and concentration distribution plot of plasma-derived exosomes. (**C**) Western blots of plasma-derived exosomes for exosome marker TSG101 as well as negative marker Grp94 carried by plasma-derived exosomes. Each lane was loaded with 5 µg protein of exosome lysate. The whole blot image can be found in Figure S1.

To analyze the cargo composition of TEX, supernatants of HNSCC cell lines, including UMSCC47, were initially used to isolate TEX. All EVs isolated by SEC from the tumor cell line supernatants are TEX. As previously described by us, the isolated small EVs in Fraction #4 meet the criteria established for exosomes regardless of HPV status of primary cells [18,20]. Specifically, Western blots of TEX show the presence of the exosome marker TSG101, as well as the absence of the negative marker Grp94 (Figure 2C). Particle sizes ranged from 80 to 150 nm as analyzed by qNano (Figure 2B) and Fraction #4 exosomes showed the typical size and vesicular morphology as visualized by TEM (Figure 2A).

**Figure 3:**
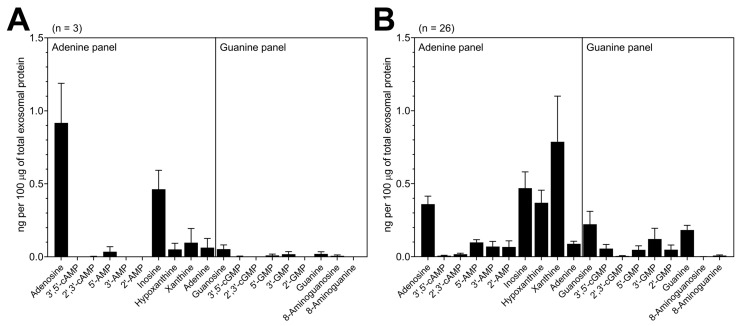
Exosomes isolated from the supernatant of HNSCC cells or patients’ plasma encapsulate purine metabolites. (**A**) Quantification of a selected panel of purine metabolites by UPLC-MS/MS in TEX derived from UMSCC47 cells. Results are presented in ng normalized to 100 μg of total exosomal protein. Presented are the means ± SEM of three independent experiments. (**B**) Quantification of purine metabolites by UPLC-MS/MS in exosomes isolated from the plasma of HNSCC patients. Results are presented in ng normalized to 100 μg of total exosomal protein. Purine metabolite levels were quantified in 26 samples from individual HNSCC patients and are here presented as means ± SEM.

The UPLC-MS/MS analysis revealed that TEX isolated from supernatants of the UMSCC47 cell line encapsulates a variety of purine metabolites. Overall, adenosine and inosine showed the highest values with 0.92 ± 0.24 and 0.46 ± 0.11 ng/100 μg of total exosomal protein (TEP), respectively (Figure 3A). Additionally, TEX carried 5′-AMP, adenine, hypoxanthine, xanthine, 3′-GMP, 5′-GMP, guanosine, guanine and 8-aminoguanosine in lower levels (Figure 3A).

### 3.3. Exosomes Derived from HNSCC Patients’ Plasma Contain Purine Metabolites

The clinicopathological data of the patients who were included in this study are listed in Table 3. In this predominantly male (69%) patient cohort, the average patient’s age was 64.2 years, representing a typical HNSCC patient population. Most tumors were located in the oral cavity (69%) followed by tumors in the larynx and pharynx. Most patients had small tumors (T1 or T2; 23% each) and 58% of the patients had negative lymph nodes (LNs). No patient had distant metastases at the time of diagnosis. Approximately half of the patients consumed alcohol (46%) and the majority (69%) consumed tobacco.

The exosomes isolated from HNSCC plasma were a mix of vesicles potentially derived from various cell types: TEX represented a variable proportion of these exosomes as we previously reported [6]. In comparison with HNSCC cell-line-derived TEX, exosomes isolated from the plasma of HNSCC patients carried a broader spectrum of purine metabolites (Figure 3B), which is not surprising due to the heterogeneous cellular origins of exosomes circulating in plasma. Most importantly, high levels of adenosine, inosine, hypoxanthine and xanthine (0.4–1.0 ng/100 μg of TEP) were detected in plasma-derived exosomes from HNSCC patients (Figure 3B). Compared to UMSCC47 cell-derived exosomes, plasma-derived exosomes showed elevated values of 2′-AMP, 3′-AMP and 5′-AMP. In addition, plasma-derived exosomes carried 3′,5′-cGMP, 2′-GMP, 3′-GMP, 5′-GMP, guanosine and guanine which were only present in low levels in TEX from UMSCC47 (Figure 3A,B).

### 3.4. Levels of Purine Metabolites are Upregulated in Plasma-Derived Exosomes from Cancer Patients Compared to Normal Donors

**Figure 4:**
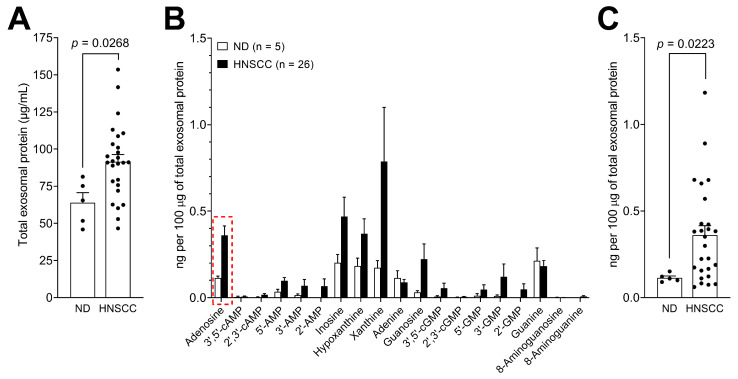
Purine metabolites are enriched in exosomes isolated from the plasma of HNSCC patients compared to those isolated from normal donors (ND). (**A**) Total exosomal protein levels (μg/mL) in samples isolated from the plasma of patients with HNSCC or ND. (**B**) Comparison of purine metabolite levels in exosomes isolated from the plasma samples of HNSCC patients or ND. Values are presented in ng normalized to 100 μg of total exosomal protein. (**C**) Individual values of adenosine levels in exosomes isolated from HNSCC or ND. Values correspond with the data marked with the red frame presented in (**B**). All values represent means ± SEM.

To determine the potential clinical significance of purine metabolite levels in plasma-derived exosomes in HNSCC, we compared exosome samples from patients (*n* = 26) with those obtained from the plasma of NDs (*n* = 5). First, TEP values were quantified and showed a significant increase in circulating exosomes in HNSCC patients compared to ND (*p* = 0.0268; Figure 4A). Next, purine metabolite levels were normalized to 100 μg TEP. Importantly, the means of almost all the purine metabolites in the panel were higher in exosomes of HNSCC patients compared to NDs (Figure 4B). Only adenine and guanine were present at similar levels in HNSCC patients and NDs. However, due to the small group numbers and a wide spread of the data in HNSCC patients, the increase for most purine metabolites was not statistically significant. Only adenosine was significantly enriched in plasma-derived exosomes from HNSCC patients compared to those isolated from the plasma of NDs (*p* = 0.0223). The individual values for adenosine in exosomes isolated from the plasma of HNSCC patients or NDs are presented in Figure 4C.

### 3.5. Purine Metabolite Levels in Plasma-Derived Exosomes are Elevated in Patients with Early-Stage Tumors

**Figure 5:**
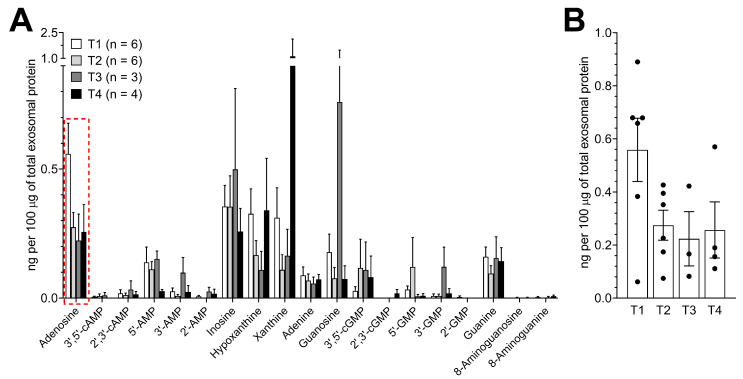
Purine metabolites levels in plasma-derived exosomes of HNSCC patients according to tumor extensions. (**A**) Values presented in Figure 3B were divided based on tumor extensions (T status) as indicated in the legend. (**B**) Individual values of adenosine levels in plasma-derived exosomes from HNSCC patients with indicated tumor extensions (T1-T4). Values correspond with the data marked with the red frame presented in (**A**). All values represent means ± SEM.

Next, the levels of purine metabolites in the panel of plasma-derived exosomes from HNSCC patients were correlated with the available clinicopathologic data. Using the tumor extension as a metric and triaging patient based on their T status, we detected no statistically significant differences in purine levels (Figure 5A). However, there was a trend for adenosine levels to decrease in exosomes from patients with increasing tumor extensions (Figure 5B).

**Figure 6:**
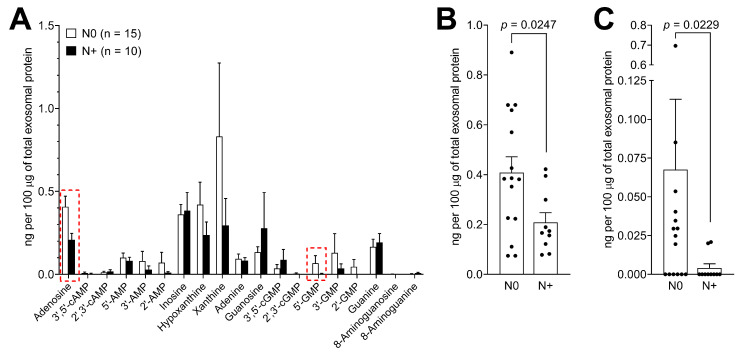
Purine metabolite levels in plasma-derived exosomes of HNSCC patients according to lymph node status. (**A**) Values presented in Figure 3B were divided based on the lymph node status (N status) and levels of purine metabolites were compared in exosomes isolated from the plasma of patients with lymph node metastasis (N+) to patients without metastasis (N0). (**B**) Individual values of adenosine levels in plasma-derived exosomes from HNSCC patients with indicated lymph node status. Values correspond with the data marked with the red frame presented in (**A**). (**C**) Individual values of 5′-GMP levels in plasma-derived exosomes from HNSCC patients with indicated lymph node status. Values correspond with the data marked with the red frame presented in (**A**). All values represent means ± SEM.

Patients were then triaged based on their lymph node (LN) involvement status and purine metabolite levels in exosomes. The exosome profiles in patients with (*n* = 10) and without (*n* = 15) LN metastasis were compared. Interestingly, most purines in the panel showed higher values in patients without LN metastasis, with only a few exceptions (inosine, 3′,5′-cGMP, guanosine, guanine; Figure 6A). Levels of adenosine were significantly decreased in exosomes of patients with LN metastasis (*p* = 0.0247; Figure 6B) as were the exosome levels of 5′-GMP (*p* = 0.0229; Figure 6C).

In aggregate, circulating exosomes of HNSCC patients with the early disease stage without LN involvement contained high levels of purine metabolites. With the disease progression and appearance of LN metastases, levels of purine metabolites in the panel decreased. Thus, changes in the purine profile of exosomes emerge as potentially useful surrogates of disease progression.

## 4. Discussion

Exosomes and their role in tumor progression are of great current interest [11,21,23]. The molecular cargo of exosomes is in part derived from the surface of parent cells and also from endosomes, providing exosomes with a molecular signature that resembles that of tumor cells [24]. The most frequently described cargo components of exosomes are proteins, nucleic acids and lipids [8]. Metabolites, which are also a part of the exosomal cargo, have received relatively little attention [25]. Studies based on different LC-MS approaches revealed that the metabolome of exosomes contained fatty acids and amino acids, steroids, prenols and eicosanoids, peptides and peptide conjugates, nucleotides, nucleosides and their derivatives, as well as less abundant sugars, alcohols, amino acids and carboxylic acids [26,27]. The knowledge of metabolites that are enriched in exosomes could be used to gain novel insights into the role of exosomes as a noninvasive liquid biopsy or as potential disease biomarkers [27].

In this study, we provided evidence that HNSCC cells have a generally accelerated purine metabolism and that exosomes from HNSCC encapsulate a broad spectrum of purine metabolites, including adenosine and its derivatives. This is a significant finding, since the stability of these metabolites as soluble circulating factors is limited. Their encapsulation into exosomes could serve as a protection against catabolism or uptake by cells. Encapsulation of purine metabolites into exosomes may also represent a mechanism for their dissemination and an opportunity to act as circulating, rather than strictly local, factors. The functional relevance of such encapsulation of purines into exosomes has been previously discussed. For example, it has been suggested that exosomes might provide an additional compartment for cAMP signaling [14]. Additionally, adenosine-rich exosomes were previously shown to promote adenosine A_2B_R-mediated angiogenesis [11]. In this study, adenosine is shown to be a prominent component of the exosome purine panel in HNSCC. Adenosine levels in exosomes of HNSCC patients were higher than those in ND’s exosomes, suggesting that tumor cells export adenosine via exosomes and utilize this mechanism to enhance autocrine protumor effects. Adenosine is an immunosuppressive and proangiogenic factor, which has a strong impact on cancer progression [5,11,13]. Adenosine byproducts, including, e.g., inosine or hypoxanthine, also have immunoregulatory activity [28]. We and others have suggested that tumor-induced dysfunction of immune cells, a common finding in HNSCC patients, is largely mediated by circulating exosomes capable of reprogramming the TME and promoting protumor activities [29]. Our experimental approach did not include the separation of TEX from non-TEX and, therefore, it is currently not clear whether purine metabolites are mainly enriched in TEX or in exosomes derived from other cell types. However, our main finding, that adenosine is the most relevant purine encapsulated in plasma-derived exosomes from HNSCC patients corresponded with our cell line-based results with pure TEX isolated from HNSCC cell lines. Additionally, we and others have reported that in patients with cancer, including HNSCC, exosome plasma levels are significantly increased relative to ND plasma [21,30]. The determination of the proportion of TEX to non-TEX in the plasma of cancer patients is a subject of ongoing research; however, based on our recent findings, TEX levels are correspondingly but variably high in patients’ plasma, and they are a major fraction of total plasma exosomes in patients with advanced disease [6]. In HNSCC, we previously showed that the suppression of lymphocyte functions by plasma-derived exosomes correlates with disease activity in patients, even without any separation of TEX and non-TEX [31].

While adenosine was the most highly and consistently enriched metabolite in TEX from tumor cell lines as well as plasma-derived exosomes in HNSCC patients, also the other purines present in the panel, including cAMP and AMP, can be readily metabolized to adenosine, increasing the total adenosine content. Additionally, noncanonical cyclic nucleotides, such as 2′,3′-cAMP were described as a source for adenosine, since extracellular 2′,3′-cAMP is metabolized to 2′-AMP and 3′-AMP, which in turn are metabolized to adenosine [32]. Similar observations were made for 2′,3′-cGMP and guanosine which were shown to increase levels of extracellular adenosine [33,34]. In contrast, it was observed that 3′,5′-cGMP, 2′,3′-cGMP, 2′,-GMP, 3′-GMP, 5′-GMP, and guanosine inhibit proliferation and induce apoptosis of cancer cells, mediating antitumor effects [35]. Interestingly, the levels of metabolites that mediate tumor progression in the adenosine panel were much higher compared to the levels of metabolites that mediate antitumor effects in the guanine panel. This suggests that TEX are selectively enriched in the cargo of metabolites that ultimately promotes tumor escape from immune surveillance and accelerates disease progression.

Interestingly, quantification of purines in exosomes of HNSCC patients by UPLC-MS/MS indicated that a cohort of patients with early disease without LN metastases had significantly elevated purine levels in circulating exosomes. Surprisingly, exosomes of HNSCC patients with stage III/IV disease and LN involvement had significantly lower purine content than that observed in the early disease with N0. Whether this finding will be reproduced in studies of exosomes in a larger cohort of patients remains to be seen, however, it might reflect an alteration in metabolic activity of parental tumor cells in patients with more advanced metastatic activity and thus might prove to have clinical significance. A lower content of purines in circulating exosomes could be an indication that purine metabolites are mainly used for cellular maintenance and proliferation in metastatic tumor cells and thus fewer are packaged into exosomes and exported outside of the cell. It seems to indicate that the molecular content of TEX and circulating exosomes is quantitatively and perhaps also qualitatively different in metastatic than in primary cancers.

Currently, it remains unresolved whether accelerated packaging of purine metabolites into exosomes in cancer is a mechanism used to eliminate an excess of “unwanted” metabolites to sustain growth and survival of cancer cells. Alternatively, purine metabolites could be shuttled by tumor cells into exosomes to mediate local and distant signaling, promoting tumor growth and inducing suppression of immune cell functions. It is possible that both mechanisms are differentially used by tumor cells, depending on the changing growth requirements during tumor progression. Future studies will be necessary to evaluate whether the additional exosome-associated purine metabolites present in exosomes, besides adenosine and cAMP, play a functional role in modulating protumor responses in the TME.

Future studies are necessary to evaluate the purine metabolite content of exosomes deriving from cells treated with inhibitors of the purine pathway, such as thiopurine drugs. Targeting the purine pathway may emerge as an additional treatment option, also with regards to our recent findings that targeting adenosine receptors impacts exosome production of cultured cells [36]. Future studies will also be necessary to analyze purine metabolites in exosomes isolated from a larger patient cohort. Ideally, these studies should be performed with matching plasma and tissue samples to evaluate possible correlations of purine metabolite levels in exosomes with the expression of markers involved in the purinergic signaling pathway in the primary tumor. Additionally, correlations of the immune phenotype and exosome cargo would be of interest to evaluate the use of exosome-associated purine metabolites as biomarkers of the immune system. These studies would serve as a basis for using exosome metabolomics as a novel noninvasive and high-throughput tool to monitor HNSCC patients.

## 5. Conclusions

This report provides the first evidence that HNSCC cells shuttle purine metabolites in exosomes, with immunosuppressive adenosine being the most prominent purine. Changes in the content and levels of purine metabolites in circulating exosomes reflect disease progression in HNSCC patients. Gaining insights into the biologic and molecular mechanisms that underlie exosome reprogramming in the TME including immune suppression provides new opportunities for future translation of exosome-based diagnostics or therapeutic interventions.

## Figures and Tables

**Table 1:** Adenine panel.

Internal Standard or Target Purine	Parent Ion (*m*/*z*)	Collision Energy (volts)	Daughter Ion (*m*/*z*)	Approximate Retention Time (min)
Analysis of Adenosine				
^13^C_10_-Adenosine	278	19	141	3.29
Adenosine	268	19	136	3.29
Analysis of 3′,5′-cAMP				
^13^C_5_-3′,5′-cAMP	335	28	136	3.8
3′,5′-cAMP	330	28	136	3.8
Analysis of 2′,3′-cAMP				
^13^C_5_-2′,3′-cAMP	335	28	136	2.42
2′,3′-cAMP	330	28	136	2.42
Analysis of 5′-AMP				
^13^C_10_-5′-AMP	358	19	141	1.72
5′-AMP	348	19	136	1.72
Analysis of 3′-AMP				
^13^C_5_-3′-AMP	353	19	136	2.16
3′-AMP	348	19	136	2.16
Analysis of 2′-AMP				
^13^C_5_-2′-AMP	353	19	136	3.10
2′-AMP	348	19	136	3.10
Analysis of Inosine				
^15^N_4_-Inosine	273	20	141	3.10
Inosine	269	20	137	3.10
Analysis of Hypoxanthine				
^13^C_5_-Hypoxanthine	141.8	22	124	1.86
Hypoxanthine	136.8	22	119	1.86
Analysis of Xanthine				
^15^N_2_-Xanthine	154.9	20	137.8	2.00
Xanthine	152.9	20	135.8	2.00
Analysis of Adenine				
^13^C_5_^15^N_5_-Adenine	146	21	128	1.50
Adenine	136	21	119	1.50

**Table 2:** Guanine Panel.

Internal Standard or Target Purine	Parent Ion (*m*/*z*)	Collision Energy (volts)	Daughter Ion (*m*/*z*)	Approximate Retention Time (min)
Analysis of Guanosine				
^13^C_10_,^15^N_5_-Guanosine	299	20	162	3.10
Guanosine	284	20	152	3.10
Analysis of 3′,5′-cGMP				
^13^C_5_-3′,5′-cGMP	351	16	152	4.28
3′,5′-cGMP	346	16	152	4.28
Analysis of 2′,3′-cGMP				
^13^C_5_-3′,5′-cGMP	351		152	4.28
2′,3′-cGMP	346	16	152	2.96
Analysis of 5′-GMP				
^13^C_10_-5′-GMP	374	15	157	1.76
5′-GMP	364	15	152	1.76
Analysis of 3′-GMP				
^13^C_10_-5′-GMP	374	15	157	1.76
3′-GMP	364	15	152	2.43
Analysis of 2′-GMP				
^13^C_10_-5′-GMP	374	15	157	1.76
2′-GMP	364	15	152	3.80
Analysis of Guanine				
^13^C_2_,^15^N-Guanine	155	20	138	1.56
Guanine	152	20	135	1.56
Analysis of 8-Aminoguanosine				
^13^C_2_,^15^N-8-Aminoguanosine	302	17	170	3.64
8-Aminoguanosine	299	17	167	3.64
Analysis of 8-Aminoguanine				
^13^C2,^15^N-8-Aminoguanine	170	18	153	1.50
8-Aminoguanine	167	18	150	1.50

**Table 3:** Clinicopathological characteristics of the HNSCC patients included in this study.

Characteristics	HNSCC Patients (*n* = 26)	
	*n*	%
*Gender*
Male	18	69
Female	8	31
*Age at diagnosis (y)*
Average ± SD	64.2 ± 13.27	
Range	39–99	
*Primary tumor site*
Larynx and pharynx	8	31
Oral cavity	18	69
*Tumor extension*		
T1	6	23
T2	6	23
T3	3	12
T4	4	15
TX	7	27
*Nodal involvement*
N0	15	58
N+	10	38
NX	1	4
*Nodal involvement*
N1	4	15
N2a	0	0
N2b	3	12
N2c	3	12
*Distant metastases*		
M0	26	100
M1	0	0
*Alcohol consumption*		
Yes	12	46
No	14	54
*Tobacco consumption*		
Yes	18	69
No	8	31

## Supplementary Materials

The following are available online at https://www.mdpi.com/2072-6694/12/6/1602/s1, Figure S1: Whole blot image of western blots shown in Figure 2C.
