# Supplementary material for: Purine Metabolites in Tumor-Derived Exosomes May Facilitate Immune Escape of Head and Neck Squamous Cell Carcinoma
Source: Cancers (Basel). 2020 Jun 17;12(6):1602. doi: 10.3390/cancers12061602 (PMC7352909; doi:10.3390/cancers12061602)
Supplement: Supplementary file 1 [file cancers-12-01602-s001.pdf]

Supplementary Materials: Purine Metabolites in Tumor-Derived Exosomes May Facilitate Immune Escape of Head and Neck Squamous Cell Carcinoma

Nils Ludwig, Delbert G. Gillespie, Torsten E. Reichert, Edwin K. Jackson and Theresa L. Whiteside

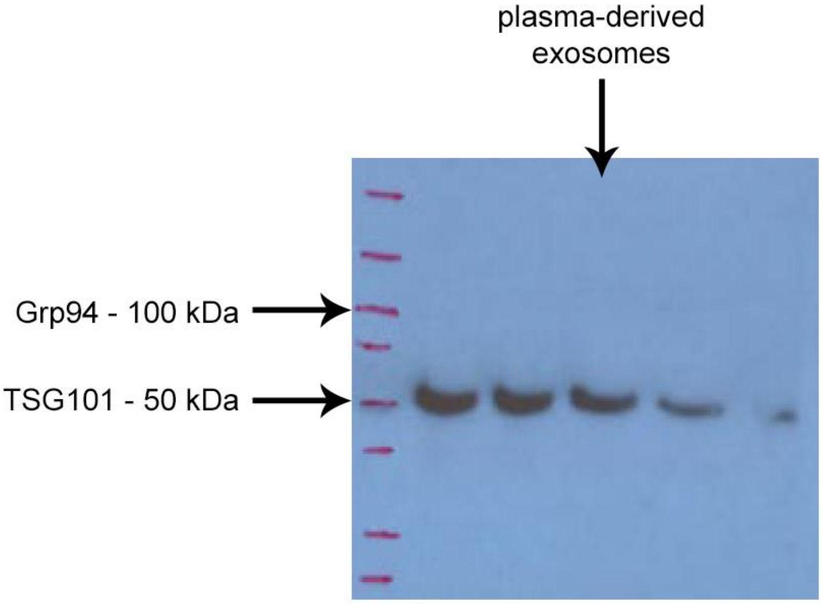

Figure S1. Whole blot image of western blots shown in Figure 2C.
